# Antithrombotic Therapy Improves ICU Mortality of Septic Patients with Peripheral Vascular Disease

**DOI:** 10.1155/2022/1288535

**Published:** 2022-03-16

**Authors:** Shiqi Yuan, Chong Chen, Fengshuo Xu, Didi Han, Rui Yang, Shuai Zheng, Mengmeng Qiao, Xiaxuan Huang, Jun Lyu

**Affiliations:** ^1^Department of Neurology, The First Affiliated Hospital of Jinan University, No. 613, Huangpu Road West, Guangzhou, Guangdong Province 510630, China; ^2^School of Public Health, Shaanxi University of Chinese Medicine, Xianyang, Shaanxi 712046, China; ^3^Department of Clinical Research, The First Affiliated Hospital of Jinan University, No. 613, Huangpu Road West, Guangzhou, Guangdong Province 510630, China; ^4^School of Public Health, Xi'an Jiaotong University Health Science Center, Shaanxi Province 710061, China

## Abstract

**Objective:**

The effectiveness of antithrombotic drugs for treating sepsis is controversial. Here, we explore the association between antithrombotic therapy and intensive care unit (ICU) mortality for septic patients with peripheral vascular disease.

**Methods:**

This retrospective cohort study uses data from the Medical Information Mart for Intensive Care (MIMIC)-III database. Kaplan–Meier survival analyses were used to examine mortality among different groups. Cox regression and marginal structural Cox models (MSCMs) were used to adjust for confounding factors. *Main Results*. The final cohort from the MIMIC-III database included 776 patients, of which 701 survived and 75 perished. The anticoagulant (AC) group and the antiplatelet-anticoagulation (AC-AP) group survived better than the group without antithrombotic treatment (non-AT). The AC and AC-AP groups showed a 0.363-fold and 0.373-fold risk of ICU mortality, respectively, compared with the non-AT group when controlling for age, gender, CRRT, alcohol, heart failure, hypertension, diabetes, obesity, renal failure, liver disease, INR, PT, PPT, and SpO2. Antiplatelet therapy did not reduce ICU mortality. The same trends were apparent from the MSCM. In addition, the AC-AP group exhibited a lower risk of bleeding complications.

**Conclusion:**

Although the antithrombotic group (AC and AC-AP groups) demonstrated a higher sequential organ failure assessment (SOFA) score than the group without antithrombotic treatment (non-AT group), the risk of ICU mortality was lower without increasing the risk of bleeding complications. Our study further suggested that anticoagulation therapy may benefit the prognosis of septic patients with peripheral vascular disease.

## 1. Introduction

Sepsis is a severe disease characterized by organ dysfunction and dysregulation of the body's inflammatory response to infection [1]. Around the world, sepsis causes almost 50 million cases with more than 11 million deaths annually [2]. Typically, sepsis is treated using antibiotic therapy, infection control, supportive care, and, in extreme cases, organ function replacement [3]. Activation of the clotting system and inflammation is necessary for the body's defense during sepsis [4]; however, septic patients generally demonstrate abnormal coagulation [3]. The three main causes of sepsis-associated coagulation dysfunction and disseminated intravascular coagulation (DIC) are coagulation activation, platelet and other inflammatory cell activation, and vascular endothelial injury [5].

While studies are currently exploring the treatment of coagulation dysfunction for sepsis recovery, anticoagulant therapy remains controversial [6–9]. Alopidogrel and clopidogrel are a widely used antiplatelet drug for cardiovascular diseases [10]. Platelet-endothelial cell and platelet-neutrophil interactions caused by platelet activation play a crucial role in microthrombosis and the release of inflammatory factors in patients with sepsis [11, 12]. However, studies on the effect of antiplatelet agents on the prognosis of patients with sepsis have produced conflicting results [13]. The conflicting efficacy of antithrombotic drugs in sepsis is likely related to the selection of the study population. Moreover, widespread peripheral vascular disease in the population increases the risk of various cardiovascular events [14–16].

In this study, we aimed to investigate the correlation between antithrombotic therapy and ICU mortality in septic patients with peripheral vascular disease.

## 2. Materials and Methods

### 2.1. Setting

The era of big data has provided unprecedented opportunities for investigating critically ill patients [17]. A large intensive care database named Intensive Care Medical Information Marketplace (MIMIC-III) was used in this study. Descriptions of MIMIC-III can be found in previous studies [18, 19]. Briefly, MIMIC-III contains data related to the hospitalizations of 53,423 adult patients (16 years of age or older) treated to the intensive care unit (ICU) between 2001 and 2012. An average of 380 laboratory measurements and 4579 chart observations were obtained for each hospitalization.

### 2.2. Participants

Infected patients with a sequential organ failure assessment (SOFA) score of at least 2 (“septic” patients as defined by the latest septic-3 diagnostic criteria) [20, 21] and peripheral vasculopathy were included our study. Infection and bleeding complications were identified using ICD-9 code, and peripheral vascular disease was screened for comorbidities. Because small number of patients may be admitted to the ICU multiple times, only patients admitted to the ICU for the first time were included in the analysis. Follow-up time was days of ICU admission, and survival status is the status at ICU discharge. The final cohort included 776 patients, of whom 701 were survivors and 75 were nonsurvivors.

### 2.3. Variable Extraction

We extracted the following patient information from the MIMIC-III database: age at admission, obesity, history of alcoholism gender, sequential organ failure assessment (SOFA) score, continuous renal replacement therapy (CRRT), history of hypertension, diabetes, renal failure, and liver disease. International normalized ratio (INR), prothrombin time (PT), partial thromboplastin time (PTT), and SpO2 were initial measurements selected for the laboratory examination.

## 3. Statistical Analysis

The study population was divided into groups with antithrombotic treatment, including antiplatelet (AP), anticoagulant (AC), and antiplatelet-anticoagulation (AC-AP) groups, and groups without antithrombotic treatment (non-AT group) based on treatment received at ICU admission. The AP, AC, and AC-AP groups include those on alopidogrel or clopidogrel, on low molecular weight heparin, and on both antiplatelet (alopidogrel or clopidogrel) and anticoagulant drugs (low-molecular-weight heparin, LMWH), respectively. The covariates of different groups were compared using an appropriate chi-square or Fisher's exact test. Continuous variables are represented by mean (standard deviation) or median (interquartile (IQR)) [22].

All statistical analyses are performed using the *R* package (version 4.1.0). Results were considered statistically significant at a *p* value of <0.05. A Kaplan–Meier survival curve analysis was used to examine differences in ICU mortality among groups (citation needed). Log-rank tests were used to compare differences among groups further (citation needed). Cox proportional risk regression models were used to analyze effects of multiple factors on survival time and status (citation needed). The marginal structural Cox model (MSCM) was used to explain the basic line, time-dependent covariates, and history of antithrombotic drug use. Antithrombotic therapy during ICU hospitalization was considered a time-dependent variable of the MSCM. The parameter of the MSCM can be estimated by taking inverse probability weighting (IPW) in the form of correcting for confusion and selection bias [23]. The package ‘IPW' was used to estimate the weight of inverse probability [24].

## 4. Results

### 4.1. Baseline Differences between Groups

The final cohort included 776 sepsis patients with peripheral vascular disease, of whom 701 were survivors and 75 were nonsurvivors.

Summary statistics of the covariates of interest for different groups are listed in Table 1. Among the different groups, differences were found in patient's SOFA score and hypertension (*p* < 0.05). The three antithrombotic treatment groups demonstrated higher SOFA scores than the non-AT group (*p* < 0.05). Among-group comparison after IPW indicated that there was no difference in these variables across treatments (*p* > 0.05) (Table S1).

### 4.2. Kaplan–Meier Survival Curve Showed the Risk of ICU Mortality among the Different Groups

**Figure 1:**
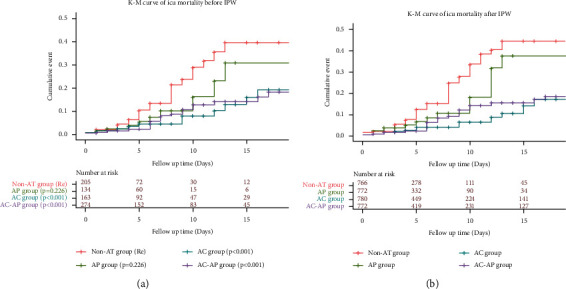
The Kaplan–Meier survival curve showing the risk of ICU mortality among the different groups ((a) before IPW, (b) after IPW). AP, antiplatelet group; AC, anticoagulant group; AC-ACP, antiplatelet-anticoagulation group. The reference group was patients that did not receive antithrombotic treatment (non-AT group). IPW, inverse probability weighting. The log-rank test was used to further compare the differences among the groups.

The antithrombotic therapy groups (AC and AC-AP groups) survived better than the non-AT group (*p* < 0.001) (Figure 1(a)). However, there was no difference in survival between AC and AC-AP groups (*p* > 0.05). We further analyzed the K–M curve after IPW, and the K–M curve for the four groups exhibited same trend compared with that without IPW (Figure 1(b)).

### 4.3. Cox Risk Regression Model Was Used to Analyze Effects of Multivariate Variables on Survival Time and Outcome

**Figure 2:**
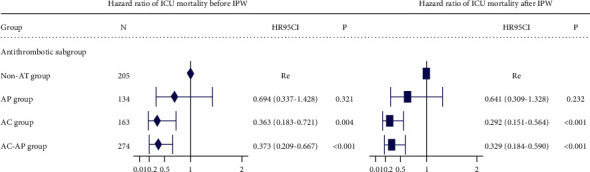
Hazard ratio (HR) of ICU mortality. On the left is the Cox proportional risk regression model before IPW and on the right is the marginal structure Cox model (MSCM) after IPW. The vertical line indicates the reference value of 1. The multivariable model was adjusted for age, gender, CRRT, alcoholism, heart failure, hypertension, diabetes, obesity, renal failure, liver disease, INR, PT, PPT, and SpO2.

We used the Cox proportional risk regression model and MSCM to investigate the effect of multivariate variables on survival time and outcomes further and estimate the hazard ratio (HR) of ICU mortality. As shown in Figure 2, the AC group showed a 0.363-fold risk of ICU mortality (HR = 0.363; 95% CI: 0.188–0.721, *p*=0.004) and the AC-AP group exhibited a 0.373-fold risk of ICU mortality (HR = 0.373; 95% CI: 0.209–0.667, *p* < 0.001) compared with the non-AT group. Similar conclusions were obtained from the MSCM analysis.

**Figure 3:**
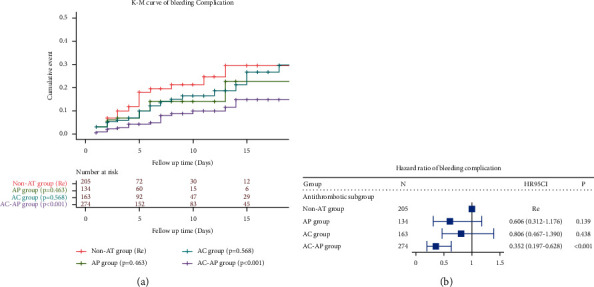
(a) A Kaplan–Meier survival curve showing the risk of bleeding complication among the different groups. (b) A Cox proportional risk regression model estimating the hazard ratio (HR) of bleeding complication. The vertical line indicated the reference value of 1. The multivariable model was adjusted for age, gender, CRRT, alcoholism, heart failure, hypertension, diabetes, obesity, renal failure, liver disease, INR, PT, PPT, and SpO2. AP, antiplatelet group; AC, anticoagulant group; and AC-AP, antiplatelet-anticoagulation group. The reference group was patients that did not receive antithrombotic treatment (non-AT group).

Bleeding emerged as a common complication of antithrombotic therapy; as such, we further studied the bleeding complications among different groups. The AC-AP group had a lower bleeding complication risk than the non-AT group (*p* < 0.001) (Figure 3(a)). Even after adjusting for multiple confounders, the AC-AP group still was at lower risk for bleeding complications (HR = 0.352; 95% CI: 0.197–0.628, *p* < 0.001) (Figure 3(b)).

Overall, our results suggested that anticoagulant therapy could substantially reduce ICU mortality without increasing the risk of bleeding complications in septic patients with peripheral vascular disease.

## 5. Discussion

The coagulation system is often activated in severe septic patients. Inflammation can incite the activation of the coagulation system, but coagulation also can activate inflammation [25]. The primary pathways leading to septicemia-induced coagulation and DIC include activation of the coagulation system, platelets, and other inflammatory cells (e.g., neutrophils and lymphocytes). Furthermore, sepsis can also cause vascular endothelial injury [5, 26], leading to multiple organ failure [27] and peripheral artery disease (PAD). Atherosclerosis is a common pathophysiological process in CAD and PAD. In atherosclerosis, vascular injury exposes the subcutaneous matrix, and platelet adhesion, activation, and aggregation (platelet-platelet and platelet-monocyte) can create lesions and lead to thrombotic complication (atherothrombosis) [28]. Therefore, sepsis-associated patients with peripheral vascular disease may be prone to abnormal coagulation.

We observed that antithrombotic therapy was associated greater survival in sepsis patients with peripheral vascular disease compared to those lacking antithrombotic therapy, even when patients without therapy were admitted to the ICU with higher incidence of organ failure than other antithrombotic therapy groups. In addition, our results suggested that anticoagulant therapy can substantially reduce ICU mortality without increasing the risk of bleeding complications for these patients. As such, our results suggest that anticoagulation therapy may improve the prognosis of septic patients with peripheral vascular disease.

This study presented the advantages of using the MSCM statistical method, considering both baseline and time-varying confusions, susceptibility of coagulation indexes of ICU patients to change, and time-dependent course of antithrombotic drug use depending on indexes of coagulation function and platelets measured in advance. Therefore, it produced a complex and dynamic relationship. The MSCM model had also been successfully applied to other time-dependent intervention studies [22, 29].

Unfortunately, the database did not provide the purpose for using low-molecular-weight heparin (LMWH). Therefore, scatter plot analysis was performed based on the dose of LMWH used by each participant. As shown in Figure S1 of supplementary materials, most participants received 5000 IU of LMWH for each dose, while a small number of participants received multiple doses during the same hospitalization (Figure S1). Although the database did not provide the purpose of LMWH use, 5000 IU daily LMWH appeared to be an appropriate prophylactic dose based on clinical practice of LMWH use.

We note the following limitations to this work. First, this study was focused on septic patients with peripheral vascular disease; however, individualized antithrombotic therapy may also improve the prognosis of patients. Second, we only examined the effects of antithrombotic drugs (alopidogrel, clopidogrel, and LMWH) while antiplatelet (indobufen, and prasugrel) and anticoagulant (rivaroxaban, and dabigatran) medications were not included in our study. As novel antithrombotic drugs in septic patients with peripheral vascular may inhibit thrombosis and bleeding risk [30, 31], prospective studies on the effects of these drugs on this pool of patients should be conducted.

## 6. Conclusion

Although the antithrombotic group (AC and AC-AP groups) demonstrated higher SOFA scores than the group without antithrombotic treatment (non-AT group), the risk of ICU mortality was lower without increasing the risk of bleeding complications. Our study suggests that anticoagulation therapy may improve the prognosis of septic patients with peripheral vascular disease and that individualized treatment may be a future direction.

## Figures and Tables

**Table 1:** Characteristics among groups before IPW. AP, antiplatelet group; AC, anticoagulant group; AC-AP, antiplatelet-anticoagulation group; and non-AT, the group that did not receive antithrombotic treatment.

Characteristics	Non-AT group	AP group	AC group	AC-AP group	*P* value
Age (median, IQR)	75.5 (66.8, 83.3)	73.6 (66.5, 80.7)	74.3 (62.5, 83)	73.7 (65.4, 81.2)	0.196
Gender (*n*, %)					0.608
** **Female	89 (43.4)	57 (42.5)	80 (49.1)	128 (46.7)	
** **Male	116 (56.6)	77 (57.5)	83 (50.9)	146 (53.3)	
CRRT (*n*, %)					0.324
** **No	198 (96.6)	130 (97)	152 (93.3)	259 (94.5)	
** **Yes	7 (3.4)	4 (3)	11 (6.7)	15 (5.5)	
Alcoholism (*n*, %)					0.054
** **No	199 (97.1)	128 (95.5)	150 (92)	266 (97.1)	
** **Yes	6 (2.9)	6 (4.5)	13 (8)	8 (2.9)	
Heart failure (*n*, %)					0.537
** **No	94 (45.9)	65 (48.5)	76 (46.6)	114 (41.6)	
** **Yes	111 (54.1)	69 (51.5)	87 (53.4)	160 (58.4)	
Hypertension					0.024
** **No	67 (32.7)	27 (20.1)	47 (28.8)	62 (22.6)	
** **Yes	138 (67.3)	107 (79.9)	116 (71.2)	212 (77.4)	
Diabetes (*n*, %)					0.319
** **No	104 (50.7)	68 (50.7)	93 (57.1)	131 (47.8)	
** **Yes	101 (49.3)	66 (49.3)	70 (42.9)	143 (52.2)	
Obesity (*n*, %)					0.175
** **No	193 (94.1)	123 (91.8)	156 (95.7)	248 (90.5)	
** **Yes	12 (5.9)	11 (8.2)	7 (4.3)	26 (9.5)	
Renal failure (*n*, %)					0.274
** **No	114 (55.6)	88 (65.7)	97 (59.5)	156 (56.9)	
** **Yes	91 (44.4)	46 (34.3)	66 (40.5)	118 (43.1)	
Liver disease (*n*, %)					0.573
** **No	190 (92.7)	123 (91.8)	145 (89)	253 (92.3)	
** **Yes	15 (7.3)	11 (8.2)	18 (11)	21 (7.7)	
Sofa (median, IQR)	4 (3, 6)	6 (4, 7)	5 (3.5, 7)	5 (3, 7.8)	0.013
INR (median, IQR)	1.2 (1.1, 1.5)	1.2 (1.1, 1.6)	1.3 (1.1, 1.8)	1.2 (1.1, 1.6)	0.668
PT (median, IQR)	13.9 (12.9, 16.1)	14.3 (12.9, 16.7)	14.5 (12.9, 19.1)	13.9 (12.8, 16.8)	0.398
PPT (median, IQR)	30.1 (26.5, 36)	32 (26.4, 39.1)	30.8 (26.9, 36.9)	30.4 (26.4, 38)	0.561
SpO2 (median, IQR)	97.2 (96, 98.5)	97.9 (96.5, 98.8)	97.7 (96.4, 98.7)	97.6 (96.2, 98.7)	0.086

## Data Availability

The MIMIC-III data were available on the project website at https://mimic.mit.edu. MIMIC database data requires database permissions, so we cannot release it to unauthorized researchers. However, if other researchers gain access to MIMIC database, we can provide the research data extracted from the MIMIC database via e-mail upon reasonable request.

## Supplementary Materials

Supplementary Materials Table S1: characteristics among groups after IPW. Figure S1: scatter plot of low-molecular-weight heparin dose per use for each participant.
