# Supplementary material for: Antithrombotic Therapy Improves ICU Mortality of Septic Patients with Peripheral Vascular Disease
Source: Int J Clin Pract. 2022 Mar 16;2022:1288535. doi: 10.1155/2022/1288535 (PMC9159174; doi:10.1155/2022/1288535)
Supplement: Supplementary Materials — Table S1: characteristics among groups after IPW. Figure S1: scatter plot of low-molecular-weight heparin dose per use for each participant. [file 1288535.f1.docx]

**Table S1:** Characteristics among groups after IPW. AP stands for antiplatelet group, AC stands for anticoagulant group, AC-AP stands for antiplatelet-anticoagulation group, and non-AT stands for the group that did not receive antithrombotic treatment.

| Characteristics | Non-AT group | AP group | AC group | AC-AP group | | *P* value |
| --- | --- | --- | --- | --- | --- | --- |
| Age (Mean, SD) | 88.23 (58.68) | 87.97 (60.20) | 87.24 (61.29) | 87.04 (59.58) | | 0.997 |
| Gender (n, %) |  |  |  |  |  | |
| Female | 344.0 (44.9) | 358.8 (46.5) | 351.1 (45.0) | 347.0 (44.9) | 0.987 | |
| Male | 421.7 (55.1) | 413.2 (53.5) | 429.4 (55.0) | 425.4 (55.1) |  | |
| CRRT (n, %) |  |  |  |  | 0.74 | |
| No | 732.7 (95.7) | 743.4 (96.3) | 731.7 (93.8) | 730.3 (94.6) |  | |
| Yes | 33.1 (4.3) | 28.7 (3.7) | 48.8 (6.2) | 42.1 (5.4) |  | |
| Alcohol (n, %) |  |  |  |  | 0.996 | |
| No | 737.4 (96.3) | 739.8 (95.8) | 749.5 (96.0) | 741.3 (96.0) |  | |
| Yes | 28.4 (3.7) | 32.2 (4.2) | 30.9 (4.0) | 31.1 (4.0) |  | |
| Heart failure (n, %) |  |  |  |  | 0.967 | |
| No | 347.4 (45.4) | 344.9 (44.7) | 334.0 (42.8) | 343.5 (44.5) |  | |
| Yes | 418.4 (54.6) | 427.2 (55.3) | 446.4 (57.2) | 428.9 (55.5) |  | |
| Hypertension |  |  |  |  | 0.932 | |
| No | 208.1 (27.2) | 195.2 (25.3) | 188.9 (24.2) | 196.5 (25.4) |  | |
| Yes | 557.7 (72.8) | 576.9 (74.7) | 591.5 (75.8) | 575.9 (74.6) |  | |
| Diabetes (n, %) |  |  |  |  | 0.992 | |
| No | 379.9 (49.6) | 395.3 (51.2) | 394.8 (50.6) | 389.5 (50.4) |  | |
| Yes | 385.9 (50.4) | 376.8 (48.8) | 385.7 (49.4) | 382.9 (49.6) |  | |
| Obesity (n, %) |  |  |  |  | 0.97 | |
| No | 712.5 (93.0) | 711.6 (92.2) | 715.6 (91.7) | 714.5 (92.5) |  | |
| Yes | 53.2 (7.0) | 60.4 (7.8) | 64.8 (8.3) | 57.8 (7.5) |  | |
| Renal failure (n, %) |  |  |  |  | 0.999 | |
| No | 444.2 (58.0) | 444.4 (57.6) | 446.5 (57.2) | 446.7 (57.8) |  | |
| Yes | 321.6 (42.0) | 327.7 (42.4) | 333.9 (42.8) | 325.7 (42.2) |  | |
| Liver disease (n, %) |  |  |  |  | 0.988 | |
| No | 705.1 (92.1) | 711.4 (92.1) | 724.4 (92.8) | 710.3 (92.0) |  | |
| Yes | 60.6 (7.9) | 60.7 (7.9) | 56.1 (7.2) | 62.1 (8.0) |  | |
| Sofa (Mean, SD) | 5.52 (3.22) | 5.54 (2.70) | 5.54 (2.73) | 5.50 (2.89) | 0.999 | |
| INR (Mean, SD) | 1.69 (1.24) | 1.65 (0.93) | 1.65 (1.49) | 1.68 (2.02) | 0.991 | |
| PT (Mean, SD) | 17.56 (10.12) | 17.42 (7.78) | 17.17 (10.82) | 16.98 (9.19) | 0.944 | |
| PPT (Mean, SD) | 37.01 (22.84) | 36.38 (20.90) | 36.62 (23.91) | 37.10 (22.08) | 0.985 | |
| SpO2(Mean, SD) | 97.12 (2.42) | 97.11 (2.43) | 97.26 (2.54) | 97.19 (2.52) | 0.953 | |


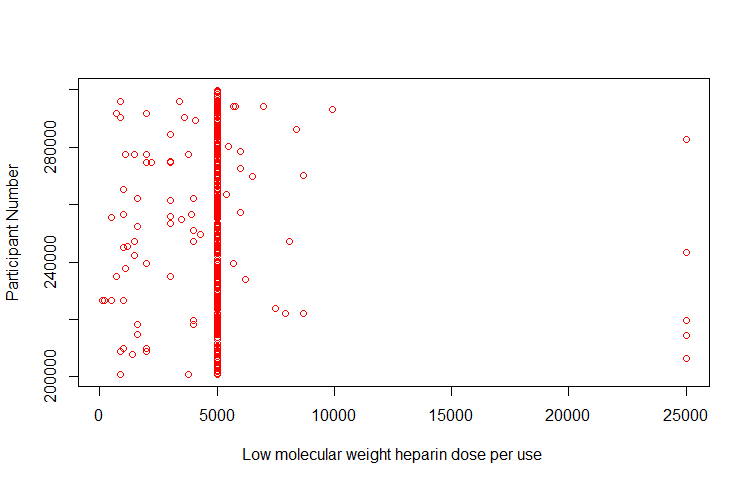


**Figure S1:** Scatter plot of low molecular weight heparin dose per use for each participant.
